# PEG Functionalization of Whispering Gallery Mode Optical Microresonator Biosensors to Minimize Non-Specific Adsorption during Targeted, Label-Free Sensing

**DOI:** 10.3390/s150818040

**Published:** 2015-07-24

**Authors:** Fanyongjing Wang, Mark Anderson, Matthew T. Bernards, Heather K. Hunt

**Affiliations:** 1 Department of Bioengineering, University of Missouri, Columbia, MO 65201, USA; E-Mail: fwwcb@mail.missouri.edu; 2 Department of Biochemistry, University of Missouri, Columbia, MO 65201, USA; E-Mail: meawm4@mail.missouri.edu; 3 Department of Chemical Engineering, University of Missouri, Columbia, MO 65201, USA; E-Mail: bernardsm@missouri.edu

**Keywords:** optical microresonator, surface functionalization, non-specific adsorption, surface characterization, PEG, nonfouling surfaces

## Abstract

Whispering Gallery Mode (WGM) optical microresonator biosensors are a powerful tool for targeted detection of analytes at extremely low concentrations. However, in complex environments, non-specific adsorption can significantly reduce their signal to noise ratio, limiting their accuracy. To overcome this, poly(ethylene glycol) (PEG) can be employed in conjunction with appropriate recognition elements to create a nonfouling surface capable of detecting targeted analytes. This paper investigates a general route for the addition of nonfouling elements to WGM optical biosensors to reduce non-specific adsorption, while also retaining high sensitivity. We use the avidin-biotin analyte-recognition element system, in conjunction with PEG nonfouling elements, as a proof-of-concept, and explore the extent of non-specific adsorption of lysozyme and fibrinogen at multiple concentrations, as well as the ability to detect avidin in a concentration-dependent fashion. Ellipsometry, contact angle measurement, fluorescence microscopy, and optical resonator characterization methods were used to study non-specific adsorption, the quality of the functionalized surface, and the biosensor’s performance. Using a recognition element ratio to nonfouling element ratio of 1:1, we showed that non-specific adsorption could be significantly reduced over the controls, and that high sensitivity could be maintained. Due to the frequent use of biotin-avidin-biotin sandwich complexes in functionalizing sensor surfaces with biotin-labeled recognition elements, this chemistry could provide a common basis for creating a non-fouling surface capable of targeted detection. This should improve the ability of WGM optical biosensors to operate in complex environments, extending their application towards real-world detection.

## 1. Introduction

Biosensors combine biological components with traditional physicochemical detection systems that operate via optical, electrical, or mechanical signal transduction mechanisms, offering advantages in the specific and timely detection of biomolecular species. Optical biosensors can be classified into two types of sensors: labeled optical biosensors, such as the fluorescence-based family of biosensors, and label-free optical biosensors, such as the refractometric family of biosensors. Labeled biosensors rely on the detection of the label, rather than the biomolecular species of interest, while label-free biosensors theoretically have a high enough signal to noise ratio (SNR) that they are capable of directly detecting the biomolecular species of interest [1]. Of these, labeled biosensors, like fluorescence-based biosensors, are the most widely-used [2], but they have a number of disadvantages including the requirements of labeling, the cost of the peripheral equipment needed to perform the detection, and the possible difficulties in conjugation and quantification due to the presence of the fluorescent label [3,4]. Label-free optical biosensors, on the other hand, may not only overcome these limitations but may also have the potential to deliver higher quality and resolution detection, with more information content and fewer false negatives, as compared to labeled biosensors [5]. Typically, these platforms use a high-sensitivity signal transducer to convert a stimulus-induced response into a quantifiable signal, without relying on dyes, enzymes, or radiolabels [5,6]. The most attractive feature of this type of biosensor is that the detection could be performed on-site and in real-time, without the need for additional peripheral equipment [5]. Due to these advantages, label-free optical biosensors have been used widely in many fields, such as medical diagnostics, drug screenings, food safety, environmental protection, biotechnology assays, and biohazard security screenings [7,8]. Moreover, they are playing an essential role in ultra-low detection studies, as well as studies designed to understand the interactions between and among biomolecular species [9,10].

**Figure 1:**
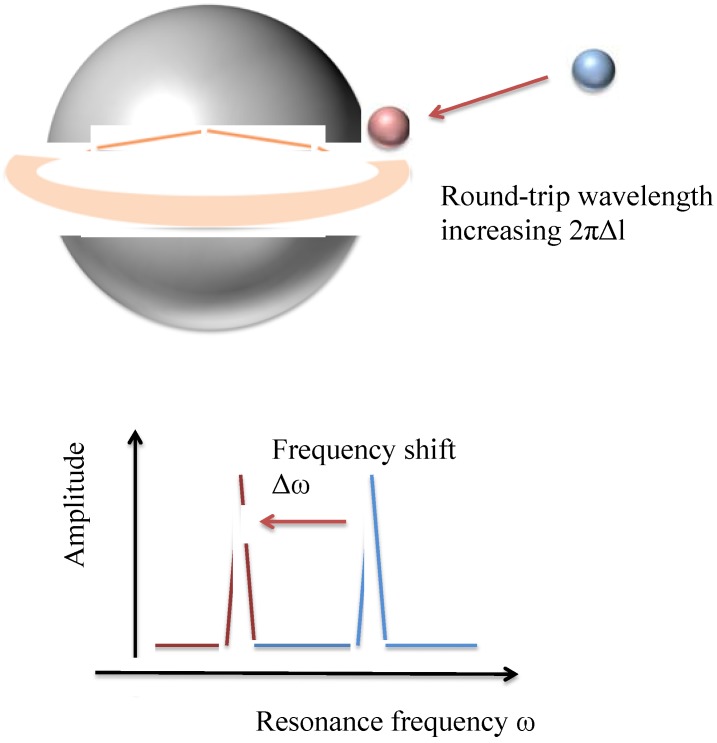
Illustration of WGM resonator, based on [17] (adapted with permission). Light (orange) enters a WGM resonator, where it experiences total internal reflection (TIR) and generates an evanescent field. When an analyte binds or adsorbs onto the surface of the microsphere, it changes the effective refractive index of the circulating optical field resonator, and it pulls part of the evanescent field to the outside of the resonator (blue for pre-binding, red for post-binding). The expansion of the optical field’s boundary causes the round-trip wavelength of light to increase about 2πΔl. The increase in the light wavelength results in a frequency shift in the transmission spectrum. The evanescent field is an optical field extending to the surrounding environment and decreasing exponentially with the distance away from the resonator’s interface.

One example of a refractometric optical device that is capable of performing label-free biosensing is the Whispering Gallery Mode (WGM) optical microresonator [11,12,13]. The Whispering Gallery Mode is a morphology-dependent resonance that has unique properties including low cavity loss [1]. To create a label-free biosensor from this device, the WGM optical microresonator must be excited via light from an external source. A necessary precondition of the occurrence of the WGM is that the dielectric optical microresonator must have a higher refractive index than the surrounding media, so that light can be spatially confined in the resonator by total internal reflection (TIR) and propagate along the microresonator’s periphery at specific resonant frequencies. The repeated reflection around the boundary of the microresonator results in the creation of an evanescent field in the surrounding environment that decreases exponentially with the distance away from the interface [14]. This field allows the microresonator to interact with biomolecules in the surrounding environment. Molecules adsorbing or binding onto the microresonator will cause a slight deviation in the effective refractive index of the circulating optical field, resulting in a detectable shift in the resonant frequency of the optical field contained by the device (Figure 1). Note that WGM positions are very sensitive to any modification of the refractive index of either the resonator or the surrounding media [15,16]. It is these interactions that give the device its sensing capabilities.

WGM optical microresonators can be fabricated in different geometries, such as microrings, microdisks, microtoroids, microspheres, microcylinders, or even asymmetric optical cavities [18,19,20,21]. However, most biosensors based on the WGM optical microcavities have been fabricated in the microsphere geometry from single-mode, silica optical fiber, typically resulting in an average diameter of 200 μm [22]. The most attractive property of the WGM optical microresonator is that both intrinsic and coupling loss can be extremely low. The total loss in the device is the primary factor in determining the maximum sensitivity of the resulting biosensor: lower intrinsic loss results in a longer photon lifetime in the microresonator. It, in turn, enhances the resulting interaction between the circulating photons and biomolecules on the microresonator’s surface [14,23]. Due to their high sensitivity, compact structure, and simple fabrication, WGM optical microresonators have been employed as an efficient platform for molecular detection, single-atom detection, and temperature measurement [24].

However, one of the primary challenges of utilizing label-free biosensors, like WGM optical microresonator-based biosensors, is the lack of a recognition element to provide specific or selective detection, in addition to sensitive detection. In order to perform either selective or specific detection, these biosensors must be combined with some kind of recognition element that allows them to selectively or specifically target a biomolecular species of interest. This recognition element could be an antibody, a protein, an enzyme, or even a functional organic group [25]. Once this task has been accomplished, the biosensor must then be capable of specific and sensitive detection in complex environments, where non-specific adsorption may occur, reducing the SNR and the overall platform performance. One of the primary challenges for label-free biosensors, and indeed, for biosensors in general, is the non-specific adsorption of unwanted biomolecules when the biosensors are used in complex environments, such as water samples, blood, and serum [1].

Although there are many techniques that may be used to reduce non-specific adsorption on biosensors, the most popular is the use of “blocker elements,” or “nonfouling elements”, as a component of the surface chemistry applied to the device to make it selective or specific. This can be done via physical adsorption, self-assembled monolayers, or covalent binding. The use of different deposition or functionalization techniques depends on the outcome desired; in many cases, the stability of a covalently-bound nonfouling element, particularly in terms of time and temperature, makes them more attractive candidates than physically adsorbed layers, despite their potential additional difficulty. Fortunately, there is also a wide variety of nonfouling elements available, allowing researchers to find a nonfouling chemistry that works best for a specific application. These include nonfouling elements, such as bovine serum albumin (BSA), lipids, non-ionic detergents like Tween 20, and of course, polymeric materials, like polyethylene glycol (PEG), as well as combinations of these elements [26,27,28,29]. PEG is one of the most-studied and general polymeric materials for nonfouling coatings, especially in the pharmaceutical, cosmetic, and biomedical fields [30]. The interest in this polymer is driven by its unique physical, chemical, and biological properties in conjunction with its behavior towards proteins and other biologically-active molecules. This includes its excellent solubility in both aqueous and organic media, and non- immunogenicity, antigenicity, or low toxicity towards living cells [31,32]. One of the most widespread applications for PEG is to resist non-specific adsorption via its strong hydration layer and steric stabilization effect [31,32,33]. This concept has been used, for instance, in biomaterials to create nonfouling surfaces, as the attachment alters the electric nature of the surface exposed to the surrounding fluids [33]. When used in conjunction with biosensor platforms, the modification by PEG is used to obtain a significant reduction in the non-specific interaction of biological molecules with the biosensor’s surface, because PEG is highly hydrophilic and has appreciable chain flexibility [34]. Non-specific adsorption decreases the SNR by increasing the background noise, and thus degenerates the sensing ability, even in high sensitivity biosensors. As mentioned above, a biosensor typically consists of a high-specificity recognition element and a high-sensitivity transducer. The PEG coatings should prevent non-specific biomolecules from binding to the surface, while allowing the recognition elements to bind with the targeted analytes, thus improving the overall performance of the biosensor.

In our previous work, we explored the use of PEG coatings in combination with WGM optical microresonator biosensors to minimize non-specific adsorption of fibrinogen and lysozyme during non-targeted detection [35]. In that work, PEG coatings of varying molecular weight were attached to the biosensor surface and were proven to have the capability to reduce non-specific adsorption. It was found that the short-chain PEG surfaces performed better in minimizing non-specific adsorption compared with long-chain PEG surfaces [35]. Here, we extend this work to targeted sensing using the biotin-avidin recognition element-target system as a proof of concept. The reasoning for the use of this system is that the biotin-avidin-biotin complex is frequently used as an intermediate sandwich complex when functionalizing surfaces of sensors; by first grafting biotin to the surface, then associating it with avidin, numerous biotin-labeled recognition elements can then be bound to the surface using the high affinity of avidin for biotin. The chemistry presented here, then, could be used as a general approach to reducing non-specific adsorption for targeted sensing using many different recognition elements. We evaluate the capability of different PEG-biotin:PEG ratios (1:1, 1:2, 1:3) in preventing non-specific adsorption, hypothesizing that the amount of exposed (PEG only) nonfouling elements would significantly impact the amount of non-specific adsorption. For each ratio chosen, the amount of the biotin recognition element (PEG-biotin) in solution was held constant while the amount of PEG nonfouling elements was increased. Fibrinogen and lysozyme were used to test for non-specific absorption to the PEG-biotin:PEG coated microresonators. The results show that these two proteins interacted minimally with the coated microresonator. Avidin was then used to test for a specific interaction. The results demonstrate that the PEG-biotin:PEG coated microresonator can effectively recognize avidin in a concentration dependent manner.

## 2. Experimental Section

**Figure 2:**
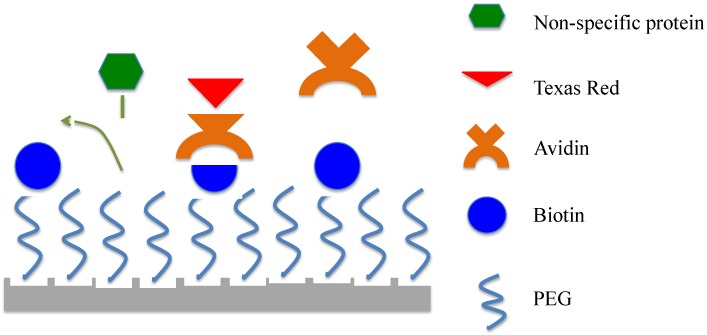
Goal: the PEG-biotin:PEG coating of the microsphere should improve the specificity by repelling non-specific protein adsortion. Avidin has four binding sites for biotin (two on each side); it is possible for avidin to bind 1–4 of these sites when it interacts with the surface, providing the biotin sites are within an appropriate distance. We expect avidin to bind to 1–2 biotin molecules on the surface, and present the other two binding sites to the environment; however, it is possible for a planar conformation to occur where all four sites bind the biotin tethered to the surface.

PEG plays an important role in resisting non-specific protein binding by creating a nonfouling surface on the microsphere. In addition, the use of biotin as the recognition element allows the creation of sandwich complexes with avidin and recognition elements, such as antibodies, proteins, receptors, *etc.*, labeled with biotin. Therefore, the combination of PEG and PEG-biotin as a coating on the microspheres should improve their specificity to the target molecules by rejecting detrimental protein molecules, and thus reducing the occurrence of false positives (Figure 2). The different ratios of PEG-biotin to PEG indicate the different densities of the nonfouling elements on the surface. We investigated if the presence of differing densities of nonfouling elements could make a significant impact on the capability of resisting non-specific adsorption.

### 2.1. Synthesis and Characterization of Functionalized (100) Silica on Silicon Wafers

To investigate the resistance of the functionalized surfaces towards non-specific adsorption, we used both (100) Si wafers with a 2 µm thermal oxide layer of SiO_2_ (University Wafers) as a control surface, as many typical surface characterization techniques have difficulty evaluating 3D curved surfaces accurately, as well as the silica microsphere optical microresonators (Section 2.2). Here, the thickness of the coating on the wafers was measured both before and after adsorption using ellipsometry. Additionally, optical profilometry and contact angle measurement were also used to investigate the surface quality and hydrophobicity characteristics. By comparing the thickness change due to the adsorption, the ratio of PEG-Biotin to PEG that demonstrated the best nonfouling characteristics could be selected and then applied to the three-dimensional optical microresonators.

**Figure 3:**
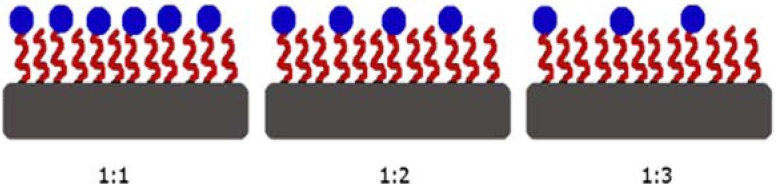
Two-dimensional schematic of the PEG-biotin:PEG ratios of 1:1, 1:2, and 1:3. The grey surface indicates the (100) silica-on-silicon wafer surfaces. The curved lines on the surface indicates the PEG coating, while the dark blue dots indicate the biotin molecules. Note that the amount of PEG-biotin in solution was kept constant, while the amount of PEG was varied.

To do this, silica-on-silicon (100) wafers (University Wafer) with a 2 µm silica (thermal oxide) grown on the surface were cut into rectangular pieces of 2 cm × 0.8 cm. Five different sets of chemistries were applied to these wafers: PEG and PEG-biotin were deposited on the wafer pieces with each of the three ratios (1:1, 1:2, and 1:3, PEG-biotin:PEG, Figure 3), and additionally, hydroxylated and biotin-only surfaces were also prepared.

**Figure 4:**
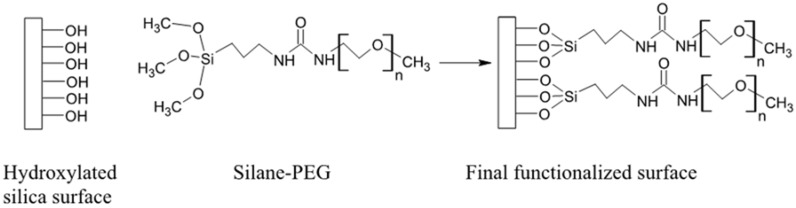
Functionalization process of silica microsphere’s surface via a two-step covalent process. Adapted with permission from [35]. Copyright (2012) American Chemical Society. The silica surface was first populated with hydroxyl groups by exposing the device to piranha solution. The hydroxylated surface was then PEG-terminated and PEG-biotin terminated using silane coupling agents attached to PEG molecules via solvent-based, covalent grafting techniques.

The PEG-biotin:PEG functionalization process was based on Soteropulos *et al.* [35]. In the first step, the silica surface was treated with piranha solution or oxygen plasma to populate the surface with terminal hydroxyl groups. Then, PEG is attached to the hydroxylated surface using a mixture of silane-PEG (2-[methoxy(polyethyleneoxy)_6-9_propyl] trimethoxysilane, MPEOPS, MW = 460–590, purity >90%, Gelest) and silane-PEG-biotin (600 Da, Nanocs) (Figure 4). The applied ratios of PEG-biotin to PEG were 1:1, 1:2, and 1:3, respectively, with the 1:1 ratio equivalent to the same PEG density in solution as the Soteropulos study using MPEOPS. Afterwards, toluene (Certified ACS, ≥99.5%, Fisher Scientific, f.w. 92.14), ethanol (Fisher Scientific, 95%) and deionized, destilled (DDI) water (Fisher Scientific, f.w. 18.02) were successively used to rinse the microspheres to remove physically adsorbed material.

**Figure 5:**
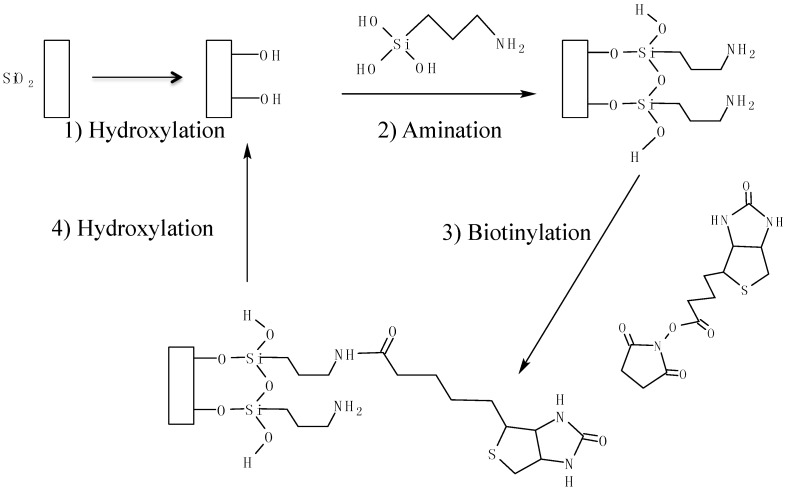
Overall reaction scheme for the biotinylation of silica surfaces (based on reference [36]). (1) Hydroxylation of the silica surface; (2) Amination of the hydroxylated surface via the silane coupling agent; (3) Biotinylation of the aminated surface via NHS ester chemistry; (4) Stripping of the surface via oxygen plasma, resulting in a hydroxylated surface.

The hydroxylated and biotin-only coatings were prepared using the protocols suggested in Hunt *et al.* [36]. The three-step conjugation is recyclable (Figure 5). First, the silica surface was terminated with hydroxyl groups with the use of oxygen plasma or piranha solution. Then, it was functionalized with 3-aminopropyltrimethoxy-silane (95%, Fisher) selectively in a vacuum desiccator for fifteen minutes. Afterwards, the surface was biotinylated with EZ-Link™ NHS-Biotin (Fisher) for thirty minutes and rinsed with DDI water. A stable amide bond was created from the NHS esters binding with the primary amines [36].

The thicknesses of the functionalized layers were then measured with a variable angle spectroscopic ellipsometer (VASE ellipsometer, J.A. Woollam, accurate to a fraction of a nm) as the initial thickness of PEG-biotin:PEG film, and were analyzed with the software Wvase32. Three randomly selected spots on each piece of wafer were measured in a wavelength range of 400–1000 nm by 10 nm increments, with angles varying from 65°–75° by 5° increments, and a dynamic averaging of 30. The measurements were taken under high accuracy mode and Isop + Depolarization sample type. To build a model fitting the data, a Cauchy layer with a calibrated thickness of 2031.336 nm was added to a 1 mm Si film (as defined in Si_jaw) to model the 2 µm silica layer of the silica-on-silicon wafer. A second Cauchy layer was added on the top to model the polymer layer. After the measurement, the wafers were then immersed in 1 mg/mL solutions of lysozyme (chicken egg white, Sigma Aldrich) or fibrinogen (bovine plasma, EMD Chemicals) in phosphate buffered saline (PBS, EMD Chemicals) for an hour, and subsequently rinsed with PBS and blown dry with nitrogen (Airgas, Ultra High Purity 5.0 Grade). The thickness measurement was taken again as the final thickness of the PEG-biotin:PEG film with non-specific adsorption, using the same settings as the initial measurement. The thickness change provides an indication of how much protein adsorption occurred on each surface. The smaller the thickness changes, the greater the film’s resistance to non-specific adsorption. The ratio minimizing protein adsorption could thus be selected.

Optical profilometry was performed to investigate the surface quality of the functionalized wafers and to compare it to the non-functionalized wafers. The nanometer-scale roughness data was obtained with an optical profiler (Veeco, WYKO NT 9109) in phase shift interferometry (PSI) mode and was analyzed with the software Vision 4.20. The data was presented with the arithmetic average of the absolute values of the roughness profile ordinates:

(1) $$R_{a}=\frac{Z_{i}-Z_{j}}{n}$$

The Contact Angle Goniometer (ramé-hart Model 200 Standard, 200-F4, accurate to 0.1°) measured the contact angle of a 2 µL DDI water drop on three randomly selected spots of each piece of wafer respectively, using the sessile drop method. The DROPimage Standard software recorded the instant contact angle for the PEG-biotin:PEG samples of three ratios, hydroxylated control, and biotin-only control. Young’s equation describes the mechanical equilibrium of the three interfacial tensions with the contact angle of a liquid drop [37]. The surface is considered hydrophilic when the contact angle is greater than 90°.

To examine if the PEG-biotin and PEG indeed deposited to the surface from the specific solution ratios, the fluorescence intensity of each sample was measured. Texas Red-Avidin was used as the fluorescent dye. Texas Red-Avidin (TR-Avidin) (Invitrogen, 2.5 mg/mL) was first diluted to 2 mg/mL with PBS and centrifuged to obtain the supernatant. The supernatant was then diluted to 10 µg/mL with PBS to incubate the functionalized wafers for 30 min in the dark, after which the wafers were rinsed with PBS and put on a cold hot plate, then warming to 40 °C for 2 min. The dye was attached to the surface due to the high affinity between biotin and avidin. Fluorescence microscopy was accomplished via an Olympus IX 70 system, using 20X magnification and the red filter. To find the best exposure time, a pseudo-color LUT (look-up table) was used to evaluate the brightness of the objects in the image during acquisition. In this LUT, the brightness of the image pixels was shown on an arbitrary scale from dark blue (black pixels with zero brightness) to white (saturated pixels with maximal brightness value 4096), as the exposure time was varied from short to long. In order to avoid saturated pixels, the exposure time was reduced until all “white” pixels were gone. This exposure time was then used for all samples. Three images, of randomly selected views, were acquired for each surface and the fluorescence intensity of four randomly selected regions of 300 nm × 300 nm in each of those images were measured with Metamorph software. Thus, twelve values of the intensity for each group (set of functionalization parameters) were obtained and then averaged during data analysis.

### 2.2. Device Fabrication, Functionalization, and Surface Characterization

Upon selecting the best ratio, the same functionalization process was applied to the silica microsphere optical microresonators. Silica microspheres with diameters of 200 μm were fabricated by melting the tip of a stripped, single-mode optical fiber (Single-mode, Newport F-SV) with CO_2_ (Synrad) laser radiation at ~8% output power [38]. The microspheres were functionalized with PEG and PEG-biotin in a 1:1 ratio via the two-step covalent attachment process shown in Figure 2. To characterize the successful deposition of the functional groups to the microresonators, both optical microscopy and fluorescence microscopy were used, with the same procedures and parameters described above.

### 2.3. Device Characterization

To fabricate the tapered optical fiber that will be used as the waveguide, a hydrogen torch was used to heat the fiber while it was stretched across a two-axis stage controller, until it reached an average waist diameter of <700 nm [1]. The microsphere and the tapered fiber were coupled to each other, monitored by optical microscopy top-view and side-view cameras.

The Quality (Q) factor is a measure of the optical performance of the microresonator. It describes the deviation from the ideal resonator and is proportional to the confinement time, or the photon lifetime, of the circulating optical field confined by the microresonator [39]. A high value of the Q factor indicates longer photon lifetime, and more interactions of the optical field with the surrounding environment. The Q factor is a direct measure of the device sensitivity. For instance, microresonators with Q factors above 10^6^ can be used for sensing single viruses [40]. In this study, the Q factor profile is recorded before and after each functionalization step, to ensure the optical microspheres’ performance does not degrade due to the synthetic modifications.

**Figure 6:**
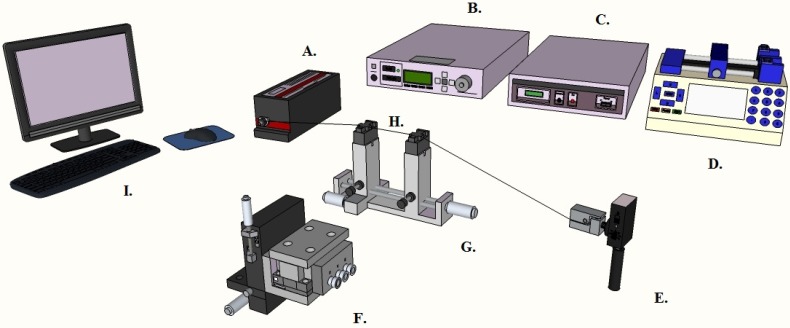
The experimental setup of device characterization. **A**—Laser **B**—Laser Controller **C**—Stage Controller **D**—Syringe Pump **E**—Photo Detector **F**—Nano-Positioning Stage **G**—Taper Holder **H**—Taper **I**—Computer. Light generated by a tunable diode laser propagates along an optical fiber. Once the Whispering Gallery Mode is excited upon coupling, the output signal is transferred to the detector and computer, and Q factor can be obtained automatically.

To do this, light from a continuous wave (CW), tunable, diode laser with a center wavelength of 980 nm (New Focus, 6320H) is introduced to a single-mode optical fiber (Newport, F-SC). The optical field is then evanescently coupled, in the undercoupled regime, to the microresonator under investigation. The under-coupled regime is more favorable than the over-coupled and the critically coupled regime, due to the minimal extrinsic loss [41]. To control the coupling distance and to attain the desired regime, the fiber was fixed while the microsphere was moved via a fiber-holder (Thorlabs) attached to a three-axis nanopositioning stage (Optosigma). The device was monitored using side- and top-view cameras simultaneously (Figure 6).

Once light was coupled into the resonator, the resonant frequency of the device could be detected via the switchable gain Si detector photodiode (Thorlabs, PDA36A). The resonance linewidth data was recorded using a digitizer/oscilloscope card directly integrated into the computer for automated data recording (NI, PCI-5153). The scan speed and direction of the laser was optimized to ensure that the resonance lineshape was not distorted. Since microspheres have multiple resonant frequencies, the frequency associated with the highest quality factor was used for the performance metrics. The resonant wavelength was recorded as λ; the peak data fitted with a Lorentzian function could give a full width at half maximum bandwidth (FWHM), denoted as Δλ. The mathematical expression for the Q factor can be summarized as: [42]

(2) $$Q=\frac{\text{λ}}{△\text{λ}}$$

### 2.4. Sensing

**Figure 7:**
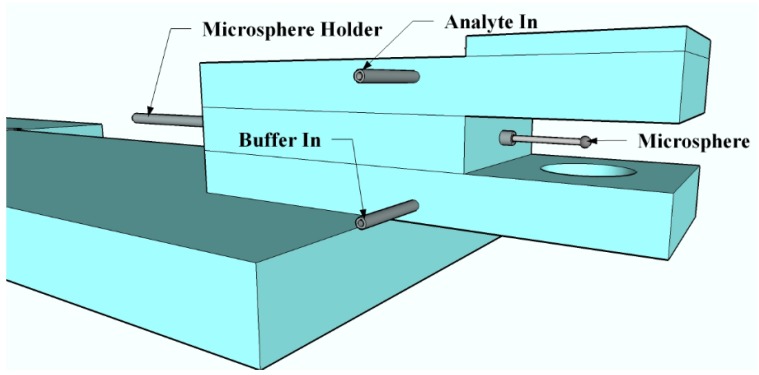
A model of the open-flow flow cell used for sensing experiments. This cell has injection ports for both the test molecule (avidin, lysozyme, or fibrinogen) and the PBS buffer solutions.

Non-specific adsorption sensing experiments, as well as avidin sensing experiments, were carried out through the use of an open-flow flow cell. The flow cell was constructed from glass slides and was fitted with metal tubing for injecting the buffer and analyte solutions as seen in Figure 7.

Sensing studies were performed using a custom LabVIEW program that located the lowest point on the oscilloscope graph of transmission voltage *versus* wavelength once the resonant wavelength with the highest quality factor was identified. The center wavelength (the lowest point on the graph) of this resonant wavelength was then tracked over time, resulting in wavelength shift data for each test molecule and concentration. For sensing experiments, the microresonator and the taper were in a coupled state. PBS buffer, which was used for the entirety of the study, was pumped up through the bottom of the flow cell using a syringe pump. The buffered microresonator-taper system was then allowed to reach equilibrium before any protein solution was added, thus allowing for the stable resonant wavelength of the system to be established. Previous literature has shown no significant resonant peak shift for control experiments completed using only PBS as the analyte [35]. A syringe pump was used to add various concentrations of the test molecule (avidin, lysozyme, or fibrinogen) into the flow cell. The PBS buffer was injected into the flow cell at a rate of 0.05 mL/min, while 0.04 mL of each test molecule solution was pumped into the flow cell at a rate of 0.03 mL/min. Solutions of lysozyme and fibrinogen, typical tests for non-specific adsorption, were created and tested at concentrations of 10, 100, 500, and 1000 μg/mL in PBS. Solutions containing avidin (Sigma Aldrich, from egg white, lyophilized powder) biotin also at concentrations of 10, 100, 500, and 1000 μg/mL, were then tested to analyze the effectiveness of the biotin recognition element and nonfouling surface that was fabricated on the microresonators.

To maintain consistency amongst the wavelength shifts and so that they were not confounded by using different microresonators, each sensing experiment was performed using the same coated microresonator. The sensing experiment consisted of evaluating fibrinogen, lysozyme, and avidin at each concentration, in that order, from the highest concentration to the lowest concentration, with a PBS rinse in between each concentration. The PBS rinse consisted of dipping the sphere in a solution of fresh PBS and placed on a rocker tray for 2 min. Then, the sphere was removed, dried, and re-rinsed using the same procedures. The entire sensing experiment was repeated two times, each time with a new, coated microresonator. Note also that the same size microresonators were used for all repeats to minimize the impact of a change in the microresonator size on the wavelength shift.

## 3. Results and Discussion

### Characterization of Functionalized Wafers

As previously introduced, the (100) silica-on-silicon wafers were functionalized with three ratios of PEG-biotin to PEG: 1:1, 1:2, 1:3, with hydroxylated and biotin-modified surfaces serving as controls. To characterize the resistance of the coated surfaces to non-specific adsorption, the thickness change due to non-specific adsorption was calculated by subtracting the thickness of the film pre-adsorption from the thickness of the film post-adsorption. A smaller thickness increase indicated a thinner protein layer attached to the surface, which in turn suggested improvement in the coating’s nonfouling characteristics.

**Figure 8:**
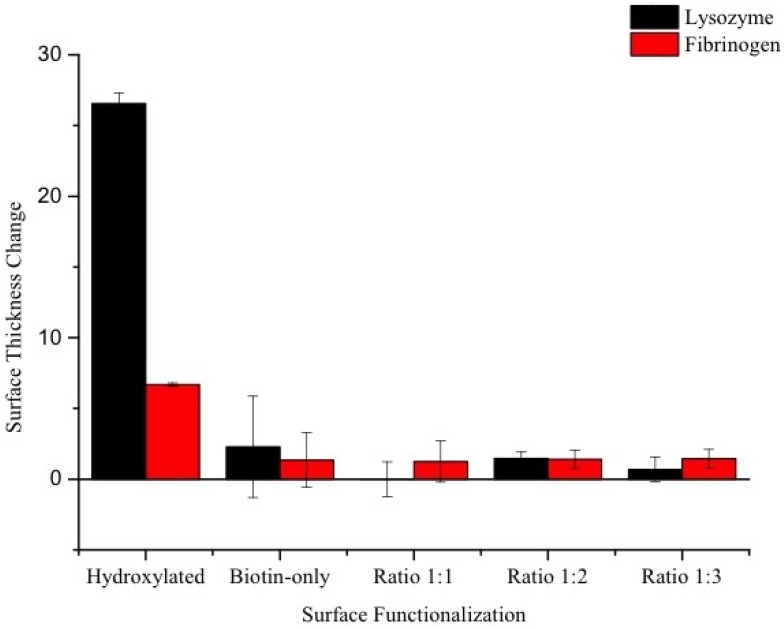
Mean thickness change (±standard deviation, nm) of functionalized wafers after adsorption. Three spots were measured on each wafer with ellipsometry, and three wafers were examined for each group. The values correspond to the amount of non-specific protein adsorbed onto the functionalized wafers.

The coatings of the as-functionalized wafers were measured with ellipsometry to be 9.7 ± 0.2 nm among the three ratios. The small standard deviation suggested that the thickness of the functionalized coatings on all the wafers, resulting from different PEG-biotin:PEG ratios, showed consistency. This measurement provided a uniform base measurement for each experiment group. After the adsorption, the thickness was recorded again. Figure 8 shows the thickness change for all three ratios and controls. It was observed that the non-specific adsorption layers on the PEG functionalized surfaces were thinner than those on the hydroxylated controls (lysozyme adsorption: 26.6 ± 0.7 nm; fibrinogen adsorption: 6.7 ± 0.1 nm), and similar to the biotin-only control (lysozyme adsorption: 2.3 ± 3 nm; fibrinogen-only adsorption: 1.4 ± 2 nm). The data further confirmed that our functionalization process indeed reduced non-specific adsorption relative to the hydroxylated control. Of greater interest is the biotin-only control (surfaces functionalized according to Figure 4), which suggests that the presence of the silane-biotin linker significantly reduces non-specific adsorption, without the need for additional nonfouling elements. However, the data from this control had a high standard deviation in comparison to the PEG-biotin:PEG surfaces, possibly due to the controls’ lower uniformity. Interestingly, all three ratios of PEG-biotin:PEG reduced non-specific adsorption in approximately equivalent amounts, although we had hypothesized that the increased density of the PEG nonfouling agents would result in a significant decrease in non-specific adsorption. This means that the lowest PEG density of the 1:1 ratio is enough to reduce the non-specific adsorption, and more PEG is not needed to reduce it further. Therefore, the ratio 1:1 was selected for application to the three-dimensional optical microresonator, which minimizes the amount of PEG needed for the functionalization. This ratio should also have the highest relative amount of PEG-biotin immobilized on the surface, leading to an increased detection capacity. It is possible that a lower PEG density would work as well as 1:1; however, our previous work functionalizing with only PEG showed that this grafting density performed best at preventing non-specific adsorption [35].

**Figure 9:**
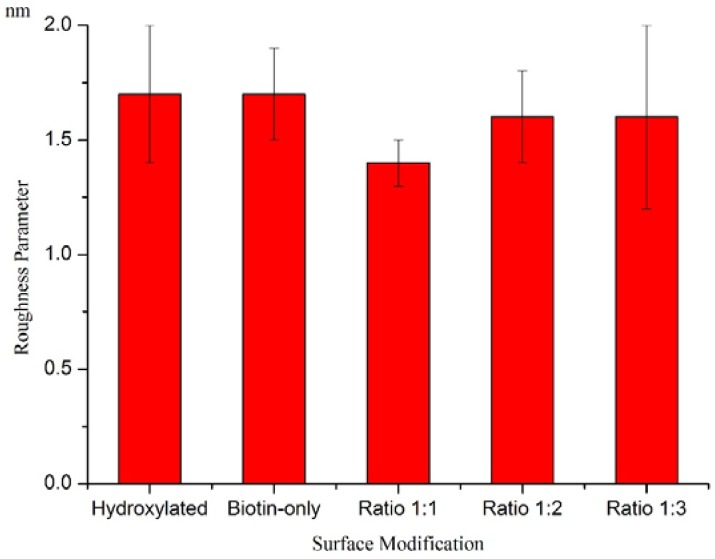
Mean Roughness Parameters (±standard deviation) of functionalized wafers after lysozyme adsorption. Three spots were measured on each wafer with optical profilometry, and three wafers were examined for each group. The addition of PEG to the surface appears to slightly reduce the overall roughness, although the initial surfaces are very smooth.

The wafers that were exposed to adsorption were also examined by optical profilometry in order to determine their surface roughness parameters. Optical profilometry was performed in PSI mode to examine the surface quality of the wafers after non-specific protein (lyzosome) adsorption. The roughness parameters were presented as the arithmetic average of the absolute values of the roughness profile ordinates. The roughness of pre-functionalization surfaces were around 1.33 nm, while that of all the post-functionalization surfaces were around 1.5 nm (Figure 9). This indicates that the post-adsorption surfaces were still very smooth.

To study the physiochemical property change of the wafers after adsorption, contact angle was measured using the sessile drop method. As Table 1 shows, both functionalized and control surfaces became less hydrophilic after the adsorption. Before non-specific adsorption, PEG-biotin:PEG coating and the molecular weight of PEG, which is around 500, both make the surface exhibit a hydrophilic character. In addition, biotin also tends to be hydrophilic due to the presence of thiol and aldehyde groups. After non-specific adsorption, the hydrophilic groups were blocked by the adsorbed protein and tend to be hydrophobic. Therefore, the less hydrophobic (or more hydrophilic) a surface is following adsorption, the less non-specific adsorption has occurred.

For example, we see a dramatic increase in contact angle of the hydroxylated control upon adsorption, which indicates the hydroxylated surface changes to be very hydrophobic. This is consistent with the ellipsometry result that the hydroxylated control shows the largest thickness increase, and this further supports the conclusion that it has the highest levels of non-specific adsorption. The other samples had lower degrees of change in the contact angle and again the results for the ratios of PEG-biotin:PEG suggested that they all performed reasonably similarly, leading to the implication that only the 1:1 ratio needed to be evaluated (the lowest density of PEG). Interestingly, the biotin-only control surface, functionalized according to Figure 4, appeared to have similar resistance to non-specific adsorption, although this was unexpected.

**Table 1:** Contact Angle Measurement before and after non-specific protein (Lysozyme and Fibrinogen) adsorption; the hydroxylated control experiences the largest increase in contact angle after adsorption, similar to the ellipsometry data, indicating that it experiences high adsorption, as expected.

Contact Angle Measurement			
	Pre-Adsorption	Post-Adsorption (L)	Post-Adsorption (F)
Hydroxylated	65.8 ± 5.0	129.2 ± 5.7	90.3 ± 3.5
Biotin-only	77.0 ± 5.7	73.1 ± 4.6	89.2 ± 4.6
Ratio 1:1	62.2 ± 2.2	74.0 ± 2.7	81.3 ± 4.1
Ratio 1:2	63.2 ± 0.8	78.5 ± 5.5	80.5 ± 3.3
Ratio 1:3	65.8 ± 2.9	90.3 ± 9.3	86.5 ± 0.5

**Figure 10:**
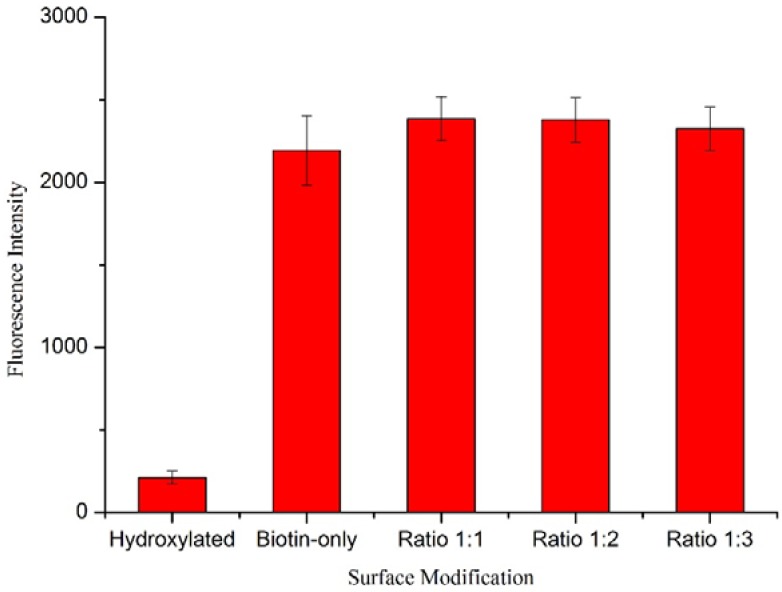
Mean fluorescence intensity (±standard deviation) of functionalized wafers. Five regions of 256 by 256 pixels were measured on each fluorescence image with an Olympus IX 70, and three images were taken for each group of wafers.

In addition, another group of wafers were functionalized with the same procedure and then were incubated in a solution of the fluorescent dye, Texas Red-Avidin, as previously described. We would expect to see a decrease in the level of fluorescence as the PEG-biotin:PEG ratio is decreased (increasing PEG) because the competitive binding levels should be relative to the solution levels as both the PEG-biotin and PEG molecules bind with identical mechanisms to the surface. Therefore, performing fluorescent intensity measurements provides information regarding the actual amount of biotin within the nonfouling surfaces. Figure 10 shows the fluorescence intensity measured with the Olympus IX 70. The data has been calibrated with a fluorescence-blank wafer subtracted from each group’s intensity. The result shows that the hydroxylated surfaces (control) gave the lowest fluorescence intensity, while all of the other surfaces showed similar levels of fluorescence. Hydroxylated surfaces showed some intensity due to a small amount of avidin chemically and/or physically adsorbed to the surface (although similar amounts of washing to remove this were used with all reactions). When biotin probe molecules were attached to the surface (Biotin-only control and all ratios), the surfaces could successfully bind more avidin than the hydroxylated surfaces, and in turn showed more fluorescence. As the PEG-biotin:PEG ratio was changed from 1:1 to 1:3, we expect a reduction in fluorescence intensity, with slight variations present due to possible effects of competitive binding. However, we note that the nonfouling surfaces resulted in nearly identical levels of, or even slightly more, Texas Red—Avidin molecules immobilized compared with biotin-only control and no noticeable differences between the ratios tested. The potential for biotin to bind multiple avidin sites should not impact non-specific adsorption, however, just fluorescence. The fluorescence results suggest that there are sufficient biotin sites present across all 3 ratios to promote a pseudo-monolayer of avidin binding. Otherwise, we would expect a decrease in the fluoresence intensity as the amount of PEG is increased. This shows that the inclusion of the nonfouling chemistry does not decrease the surface’s ability to bind with its target analyte. This further suggests there is no benefit in diluting the PEG-biotin concentration below the 1:1 ratio.

**Figure 11:**
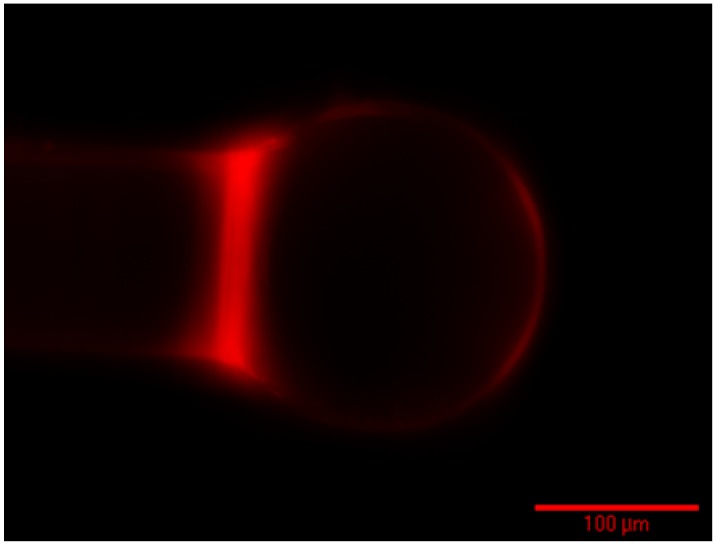
Representative fluorescence microscopy image of the microsphere functionalized by PEG-biotin:PEG from a ratio of 1:1. The functionalization process resulted in a uniform and smooth coverage on the surface.

The selected PEG-biotin:PEG ratio of 1:1 was applied to the silica microspheres. The modified spheres were divided to two groups, one was for surface quality examination, and the other was for sensing experiments. After labeling the coated spheres with Texas Red-Avidin, fluorescence microscopy was used to investigate the uniformity of the coating. Figure 11 shows an image of a functionalized microsphere. A robust and uniform coating can be observed. It indicates that the functionalization process was successful in coating microspheres with PEG-biotin:PEG solution.

**Figure 12:**
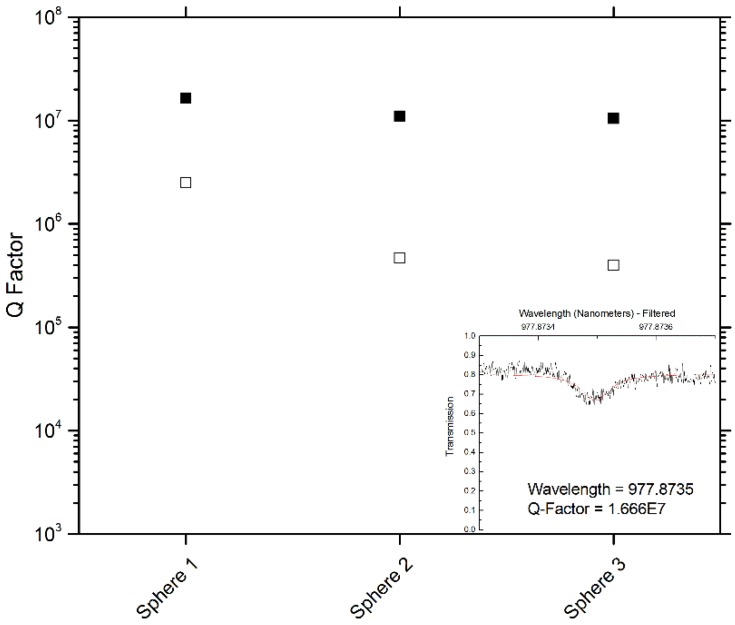
Quality factors of each microresonator used in the sensing study before (solid squares) and after (hollow squares) coating. Inset: a representative resonance (black line—data, red line—Lorentzian fit), showing a high quality factor device.

The microresonators used in this study (three total, for repetitions of sensing experiments) were evaluated for their Q factor before and after coating. Figure 12 shows these values, as well as a representative resonance, fit with a Lorentzian function. Typically, the Q factor of the silica microspheres drop an order of magnitude after coating.

**Figure 13:**
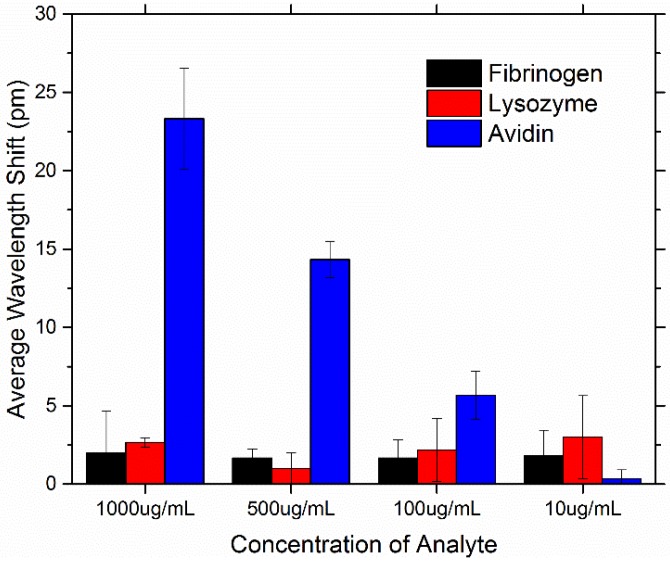
Average resonant wavelength shift of the PEG-biotin:PEG coated microresonators at concentrations of 1000, 500, 100, and 10 μg/mL for fibrinogen, lysozyme, and avidin.

**Figure 14:**
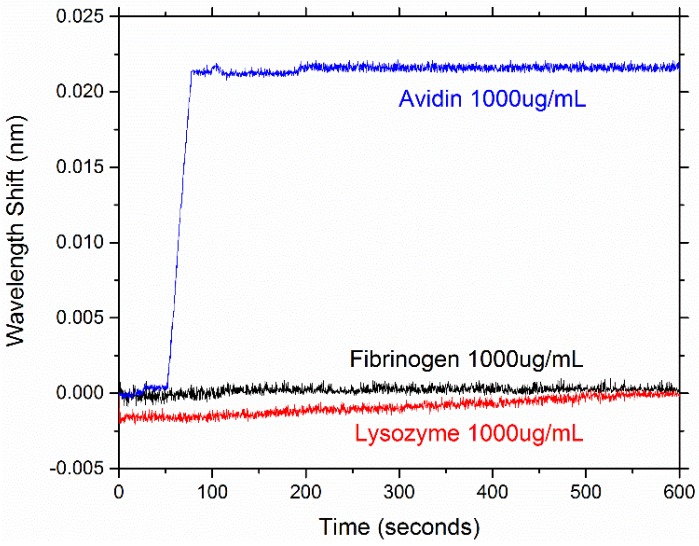
A representative example of the resonant wavelength shift of one of the PEG-biotin:PEG coated microresonators used in the study for different concentrations of fibrinogen, lysozyme, and biotin at the concentration of 1000 μg/mL. Fibrinogen and lysozyme show a minimal response, while avidin has a clearly defined wavelength shift that is approximately 20 pm.

Sensing results show that coating microresonators with PEG has effectively reduced the non-specific binding of both fibrinogen and lysozyme. Previous work has shown wavelength shifts in the range of 20–30 pm for bare (hydroxylated) microspheres [35]. Figure 13 shows the average resonant peak wavelength shift for all analytes tested at all concentrations, and Figure 14 highlights the specificity the coated microresonators have for avidin. We note that, for targeted sensing, a low limit of detection is necessary; however, for non-specific adsorption, a different metric should be evaluated to determine if the device performed well. In the case of non-specific adsorption, we typically want the surface to repel proteins, for example, at not just low concentrations but also high concentrations. Therefore, demonstrating that across a 100-fold increase in concentration, maintaining a statistically low level of background, non-specific adsorption, is important. Here, we show that not only can the surface “repel” or “block” non-specific adsorption at 10 µg/mL but also at the much more biologically relevant concentration of 1000 µg/mL.

**Figure 15:**
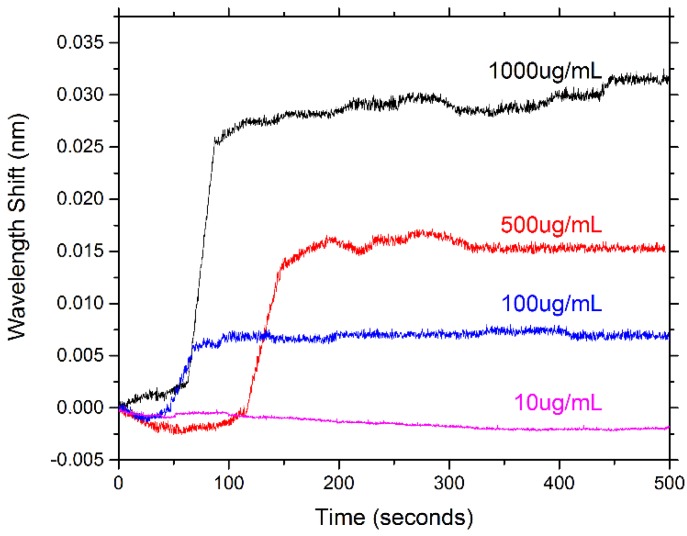
Representative response of one of the PEG-biotin:PEG coated microresonators for different concentrations of avidin. The wavelength shift declines as the concentration of avidin declines. Minimal response is seen at the concentration of 10 μg/mL.

Here, microresonators coated with PEG show an average shift in response to fibrinogen/lysozyme in the range of 0–5 pm. The sensing results with respect to avidin demonstrate that the coated microresonators can also recognize avidin in a concentration dependent manner. At avidin’s highest concentration tested (1000 µg/mL), wavelength shifts in the range of 20–30 pm were seen. However, at avidin’s lowest concentration tested (10 µg/mL), no significant wavelength shift was seen. Figure 15 illustrates the concentration dependent response of biotin for a given microresonator. The results in this study indicate that PEG-biotin:PEG coated microresonators may be able to effectively recognize avidin in a complex environment where non-specific proteins are present.

## 4. Conclusions/Outlook

In this study, we have demonstrated the nonfouling characteristics of PEG when coated on the surface of a Whispering Gallery Mode optical microresonator that is also functionalized with recognition elements (PEG-biotin). The reasoning for the use of this system, and, in particular, the use of biotin as a “recognition element”, is that the biotin-avidin-biotin complex is frequently used as an intermediate sandwich complex when functionalizing surfaces of sensors. By first grafting biotin to the surface, then associating it with avidin, numerous biotin-labeled recognition elements can then be bound to the surface using the high affinity of avidin for biotin. The chemistry presented here, then, could be used as a general approach to reducing non-specific adsorption for targeted sensing using many different recognition elements. We evaluated the capability of different PEG-biotin:PEG ratios (1:1, 1:2, 1:3) in preventing non-specific adsorption, hypothesizing that the amount of exposed (PEG only) nonfouling elements would significantly impact the amount of non-specific adsorption. For each ratio chosen, the amount of the biotin recognition element (PEG-biotin) in solution was held constant while the amount of PEG nonfouling elements were increased. Fibrinogen and lysozyme were used to test for non-specific absorption to the PEG-biotin:PEG coated microresonators. The results showed that fibrinogen and lysozyme had minimal interactions with the coated wafers and microresonators, in comparison to the hydroxylated controls, and that increasing the PEG density on the surface did not significantly reduce non-specific adsorption beyond the 1:1 ratio. More interestingly, we found via the biotin-only control, which was functionalized with a simple silane coupling agent instead of PEG, yielded potentially comparable results to the PEG-biotin:PEG surfaces. This is surprising, as the silane coupling agent and the silane coupling agent used (aminopropyltrimethoxysilane, APTMS) are not known for their nonfouling properties in this sense. Avidin was then used to test for a specific interaction, and showed that the coated resonators were still capable of performing concentration-dependent detection. The combination of recognition and nonfouling elements should provide a means to increase the specificity of optical sensing by reducing the noise caused by non-specific adsorption. The covalently-bound nonfouling and recognition elements provide a means to increase the specificity of optical sensing by reducing the noise from non-specific adsorption. Due to the frequent use of biotin-avidin-biotin sandwich complexes in functionalizing sensor surfaces with biotin-labeled recognition elements, this chemistry could provide a common basis for creating a non-fouling surface capable of targeted detection. This should improve the ability of WGM optical biosensors to operate in complex environments, extending their application towards real-world detection.
